# Supplementary material for: Endometriosis and Impaired Placentation: A Prospective Cohort Study Comparing Uterine Arteries Doppler Pulsatility Index in Pregnancies of Patients with and without Moderate-Severe Disease
Source: Diagnostics (Basel). 2022 Apr 19;12(5):1024. doi: 10.3390/diagnostics12051024 (PMC9139463; doi:10.3390/diagnostics12051024)
Supplement: Supplementary file 1 [file diagnostics-12-01024-s001.zip › diagnostics-1644597-supplementary.pdf]

Figure S1. Average marginal effects with 95% confidence intervals (CIs) for all the independent variables included in the general linear model (GLM).

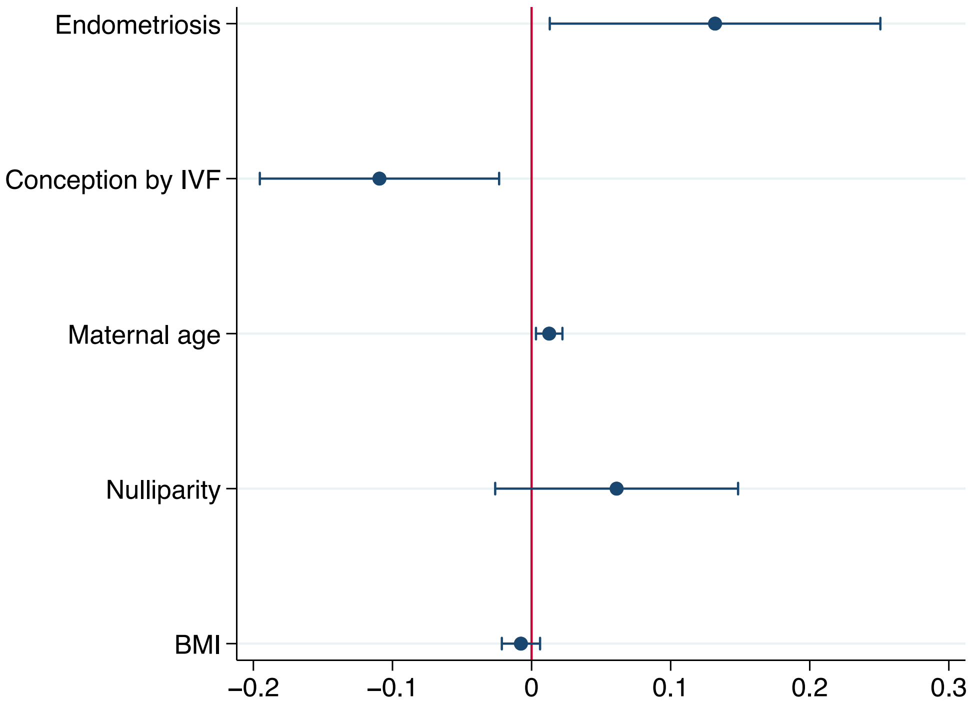

Notes: Variables included in the GLM are reported on the y-axis; average marginal effects with 95% CIs (i.e., effect on the linear prediction) are reported on the x-axis. Dots represents the beta-coefficient for each variable, whereas continuous horizontal lines represent 95% CI. The shorter is the 95% CI the more precise is the effect of the explanatory variable on the linear estimate of the response variable. Predictors with 95% CI crossing the null value (red vertical line) did not show a significant effect on the linear prediction of third trimester UtA-PI Z-scores in the multivariable model.

Abbreviations: UtA-PI, uterine artery pulsatility index; IVF, in vitro fertilization; BMI, body mass index.
